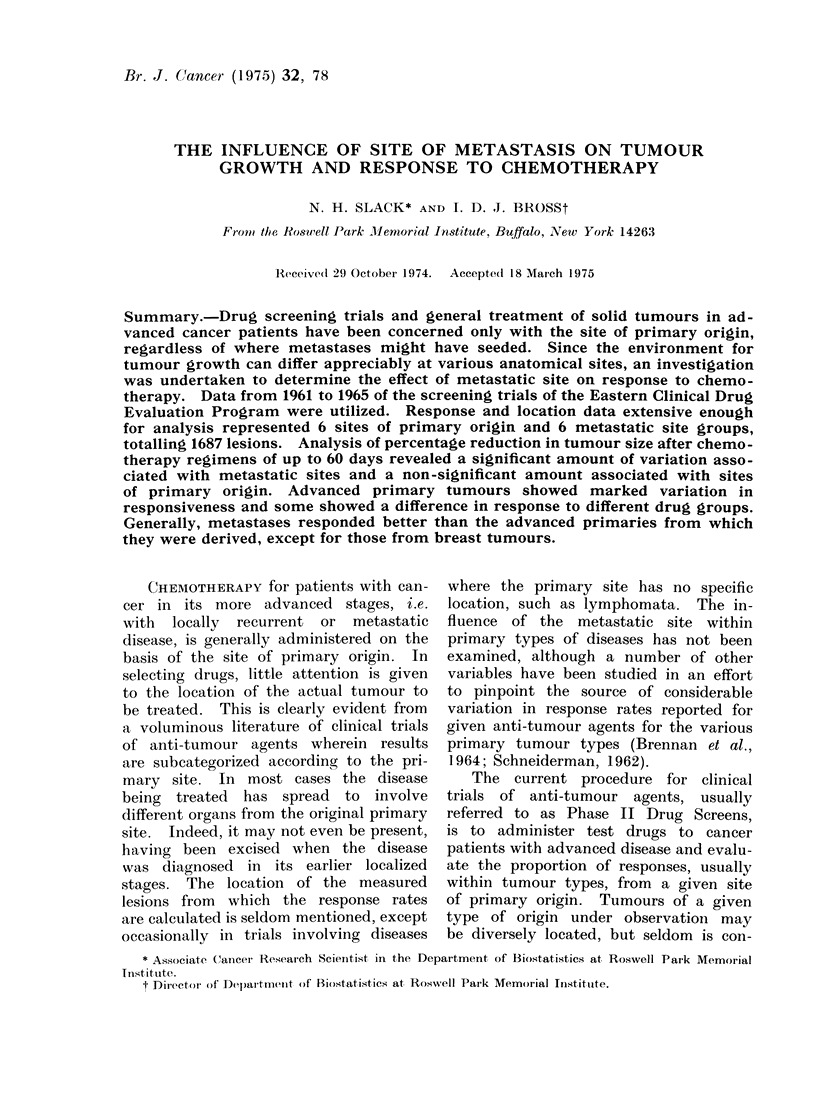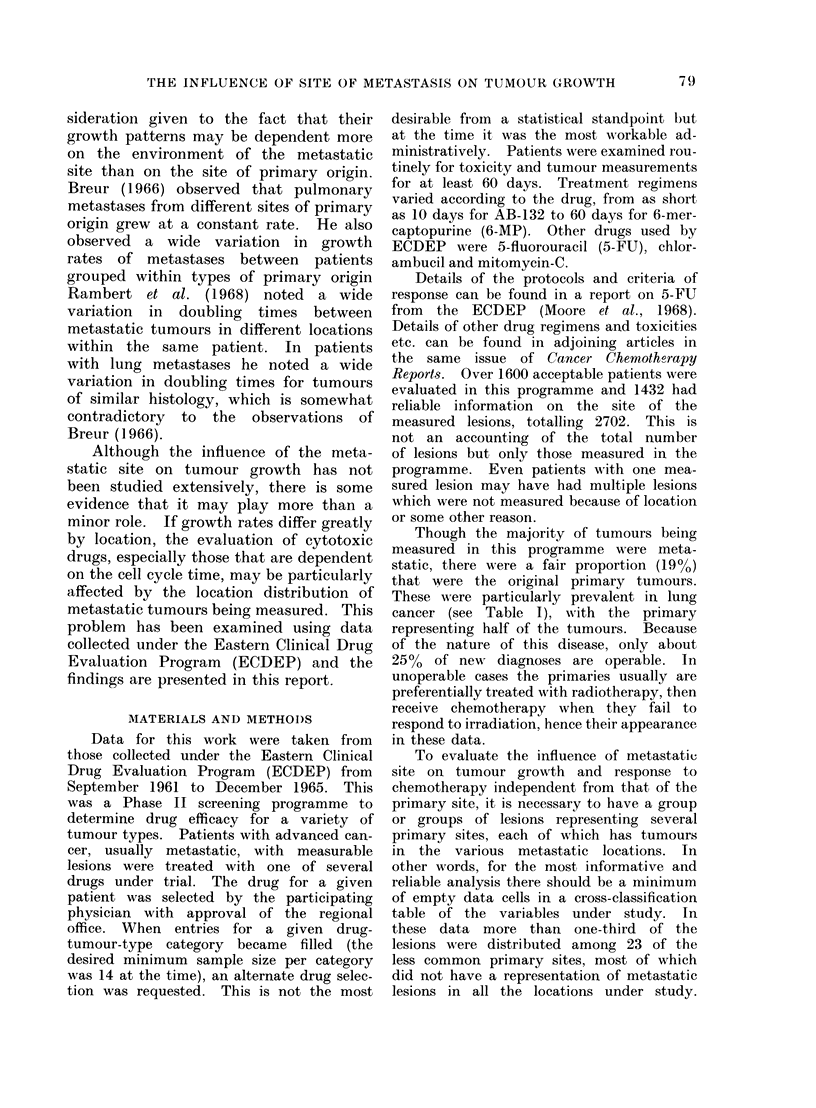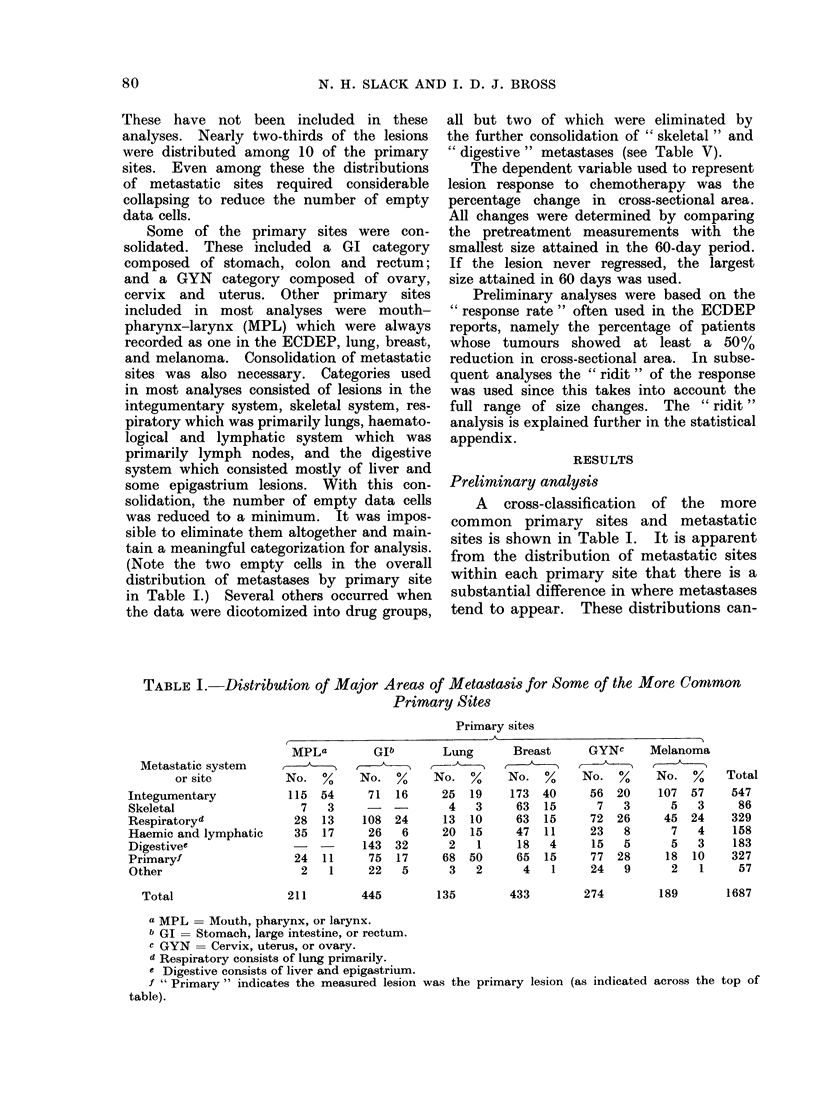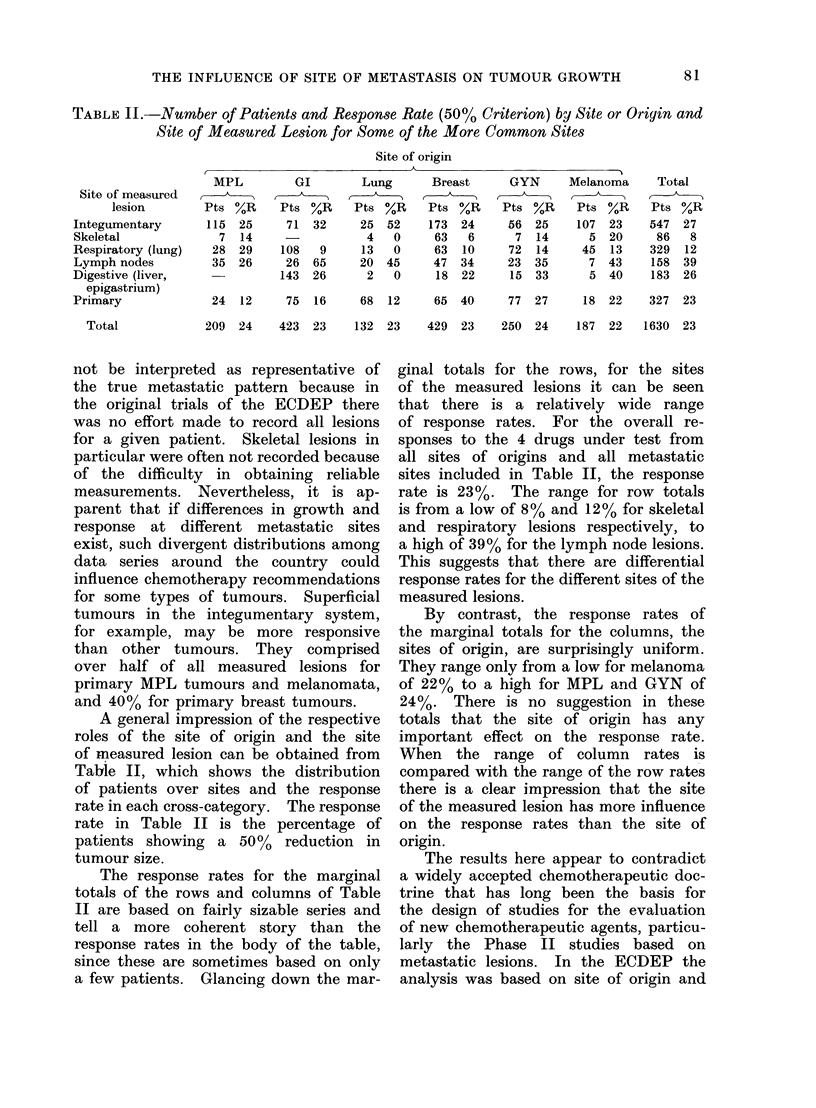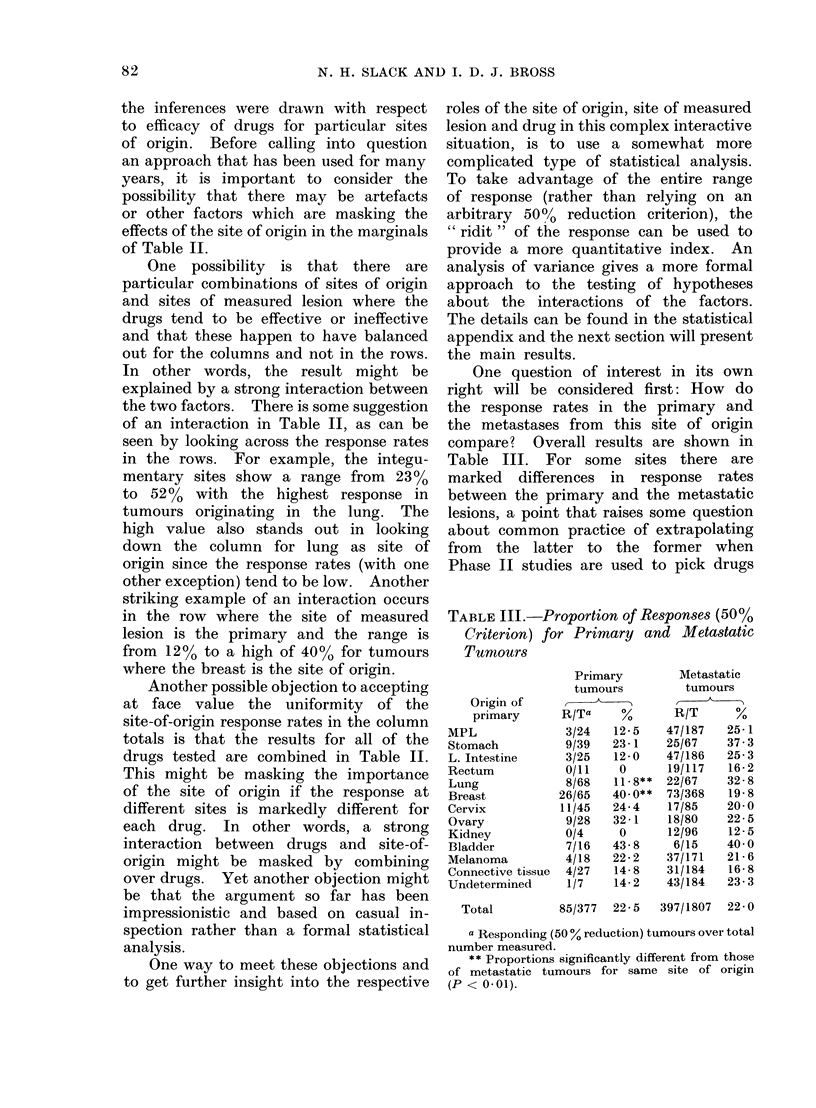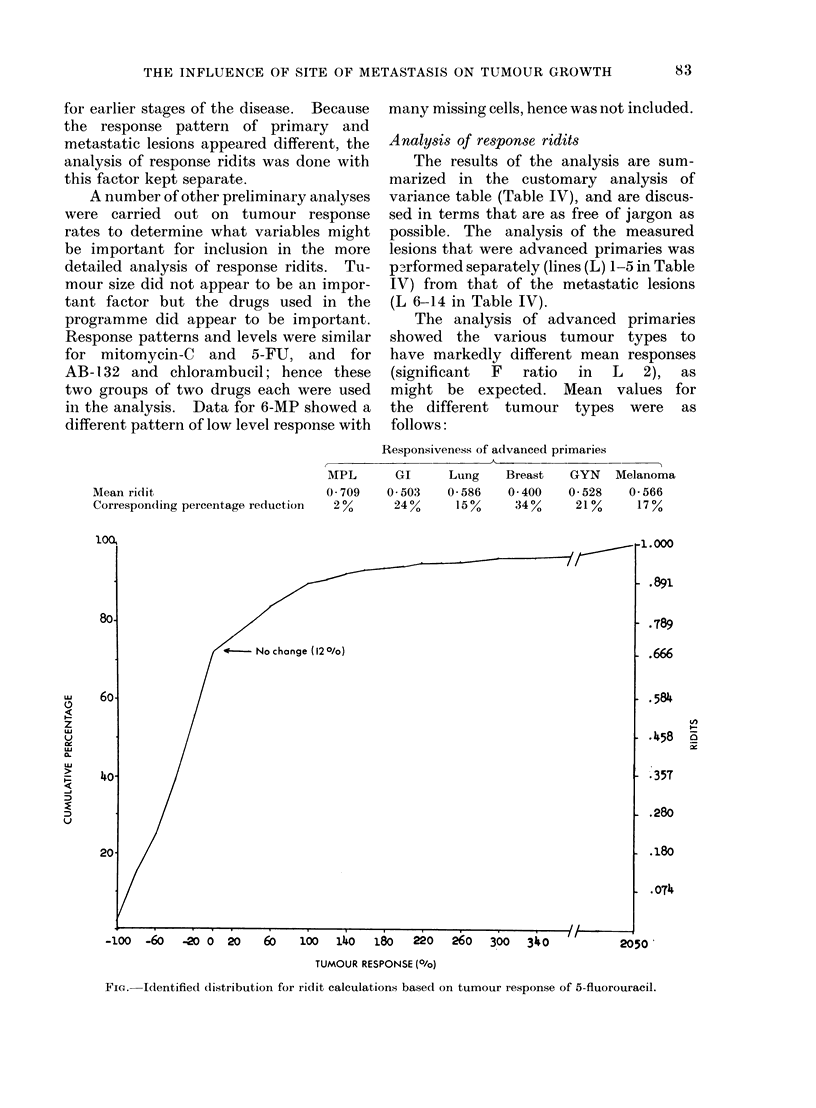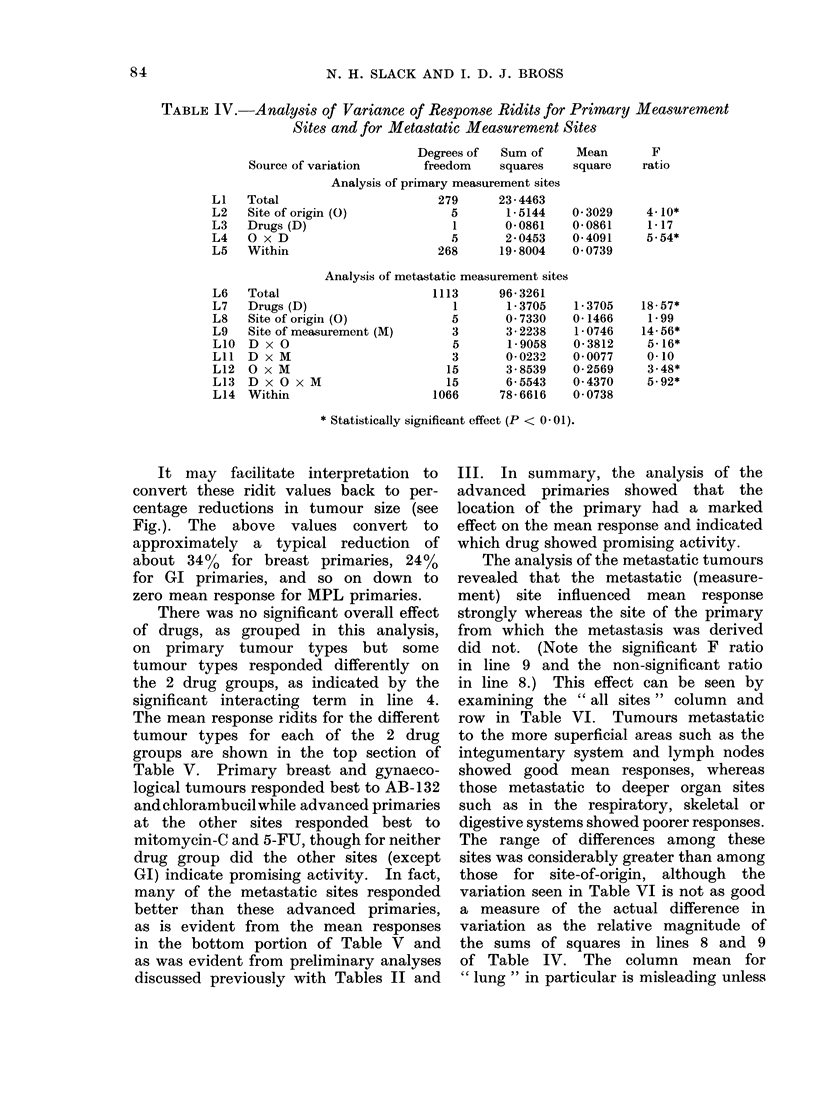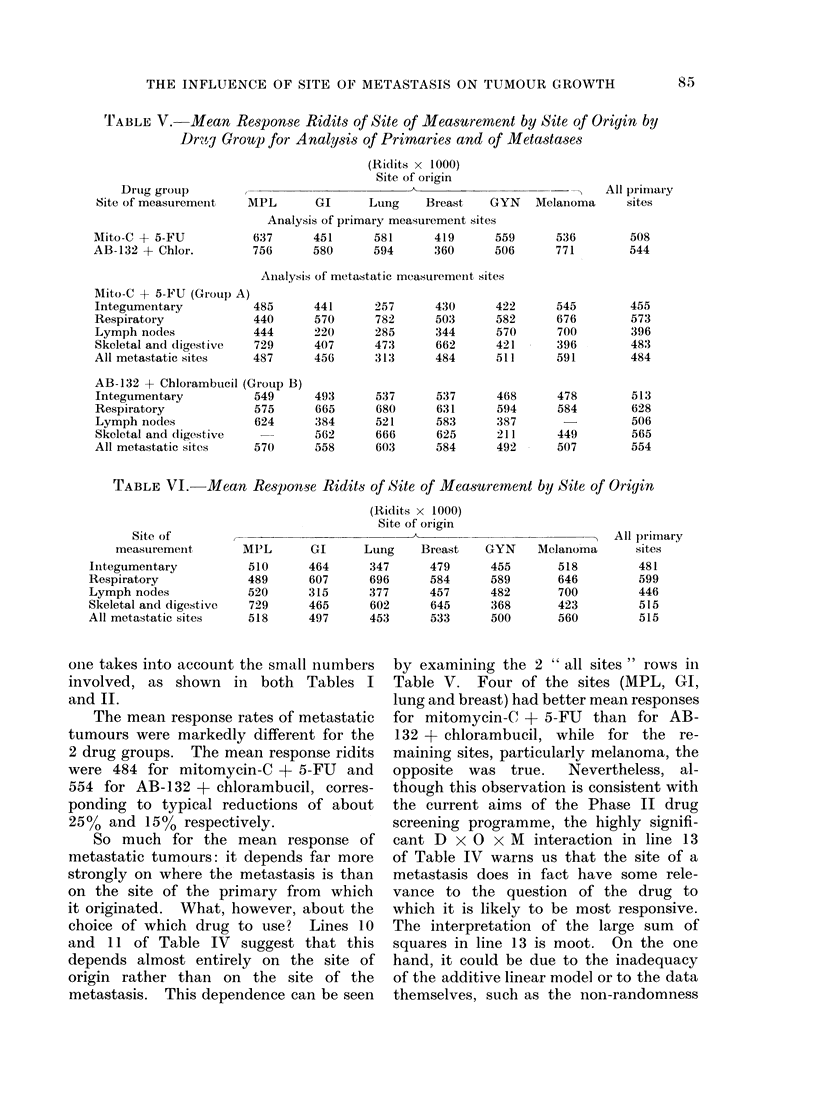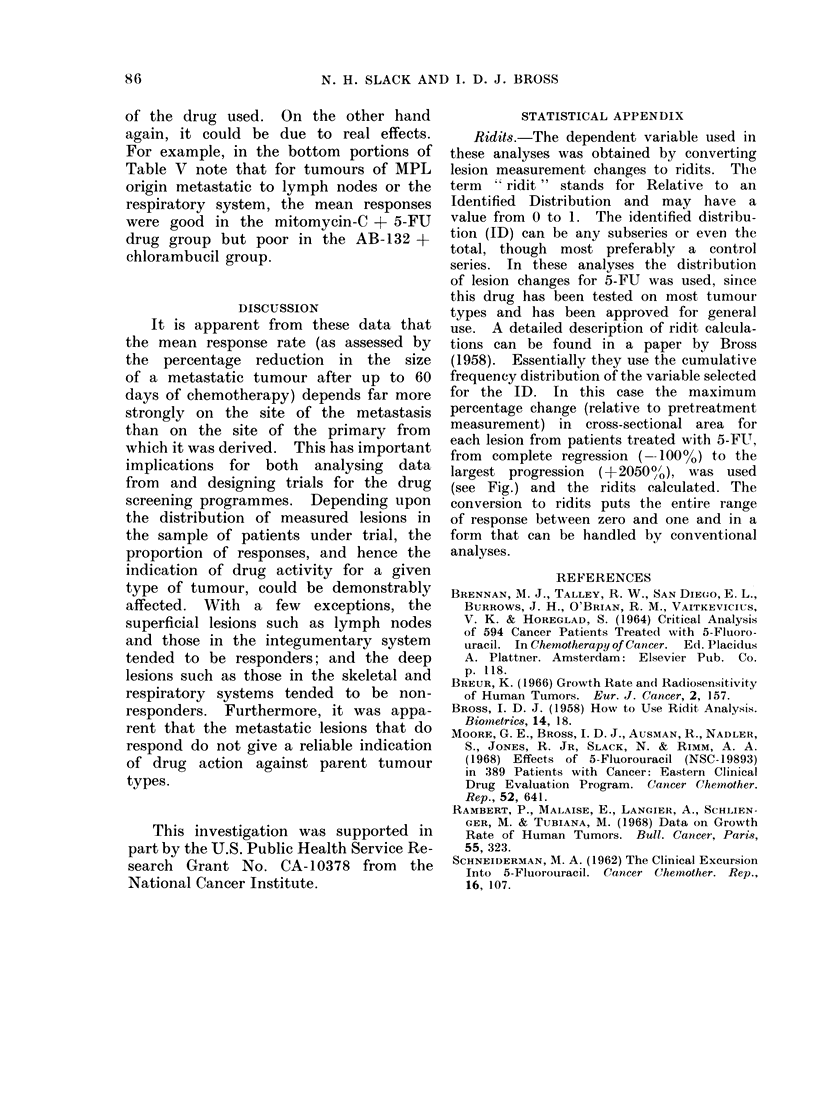# The influence of site of metastasis on tumour growth and response to chemotherapy.

**DOI:** 10.1038/bjc.1975.135

**Published:** 1975-07

**Authors:** N. H. Slack, I. D. Bross

## Abstract

Drug screening trials and general treatment of solid tumours in advanced cancer patients have been concerned only with the site of primary origin, regardless of where metastases might have seeded. Since the environment for tumour growth can differ appreciably at various anatomical sites, an investigation was undertaken to determine the effect of metastatic site on response to chemotherapy. Data from 1961 to 1965 of the screening trials of the Eastern Clinical Drug Evaluation Program were utilized. Response and location data extensive enough for analysis represented 6 sites of primary origin and 6 metastatic site groups, totalling 1687 lesions. Analysis of percentage reduction in tumour size after chemotherapy regimens of up to 60 days revealed a significant amount of variation associated with metastatic sites and a non-significant amount associated with sites of primary origin. Advanced primary tumours showed marked variation in responsiveness and some showed a difference in response to different drug groups. Generally, metastases responded better than the advanced primaries from which they were derived, except for those from breast tumours.


Br. J. Cancer (1975) 32, 78

### THE INFLUENCE OF SITE OF METASTASIS ON TUMOUR GROWTH AND RESPONSE TO CHEMOTHERAPY

N. H. SLACK* AND I. D. J. BROSS†

*From the Roswell Park Memorial Institute, Buffalo, New York 14263*

Received 29 October 1974. Accepted 18 March 1975

**Summary.—Drug screening trials and general treatment of solid tumours in advanced cancer patients have been concerned only with the site of primary origin, regardless of where metastases might have seeded. Since the environment for tumour growth can differ appreciably at various anatomical sites, an investigation was undertaken to determine the effect of metastatic site on response to chemotherapy. Data from 1961 to 1965 of the screening trials of the Eastern Clinical Drug Evaluation Program were utilized. Response and location data extensive enough for analysis represented 6 sites of primary origin and 6 metastatic site groups, totalling 1687 lesions. Analysis of percentage reduction in tumour size after chemotherapy regimens of up to 60 days revealed a significant amount of variation associated with metastatic sites and a non-significant amount associated with sites of primary origin. Advanced primary tumours showed marked variation in responsiveness and some showed a difference in response to different drug groups. Generally, metastases responded better than the advanced primaries from which they were derived, except for those from breast tumours.**

CHEMOTHERAPY for patients with cancer in its more advanced stages, *i.e.* with locally recurrent or metastatic disease, is generally administered on the basis of the site of primary origin. In selecting drugs, little attention is given to the location of the actual tumour to be treated. This is clearly evident from a voluminous literature of clinical trials of anti-tumour agents wherein results are subcategorized according to the primary site. In most cases the disease being treated has spread to involve different organs from the original primary site. Indeed, it may not even be present, having been excised when the disease was diagnosed in its earlier localized stages. The location of the measured lesions from which the response rates are calculated is seldom mentioned, except occasionally in trials involving diseases where the primary site has no specific location, such as lymphomata. The influence of the metastatic site within primary types of diseases has not been examined, although a number of other variables have been studied in an effort to pinpoint the source of considerable variation in response rates reported for given anti-tumour agents for the various primary tumour types (Brennan *et al.*, 1964; Schneiderman, 1962).

The current procedure for clinical trials of anti-tumour agents, usually referred to as Phase II Drug Screens, is to administer test drugs to cancer patients with advanced disease and evaluate the proportion of responses, usually within tumour types, from a given site of primary origin. Tumours of a given type of origin under observation may be diversely located, but seldom is con-

* Associate Cancer Research Scientist in the Department of Biostatistics at Roswell Park Memorial Institute.

† Director of Department of Biostatistics at Roswell Park Memorial Institute.

sideration given to the fact that their growth patterns may be dependent more on the environment of the metastatic site than on the site of primary origin. Breur (1966) observed that pulmonary metastases from different sites of primary origin grew at a constant rate. He also observed a wide variation in growth rates of metastases between patients grouped within types of primary origin Rambert *et al.* (1968) noted a wide variation in doubling times between metastatic tumours in different locations within the same patient. In patients with lung metastases he noted a wide variation in doubling times for tumours of similar histology, which is somewhat contradictory to the observations of Breur (1966).

Although the influence of the metastatic site on tumour growth has not been studied extensively, there is some evidence that it may play more than a minor role. If growth rates differ greatly by location, the evaluation of cytotoxic drugs, especially those that are dependent on the cell cycle time, may be particularly affected by the location distribution of metastatic tumours being measured. This problem has been examined using data collected under the Eastern Clinical Drug Evaluation Program (ECDEP) and the findings are presented in this report.

### MATERIALS AND METHODS

Data for this work were taken from those collected under the Eastern Clinical Drug Evaluation Program (ECDEP) from September 1961 to December 1965. This was a Phase II screening programme to determine drug efficacy for a variety of tumour types. Patients with advanced cancer, usually metastatic, with measurable lesions were treated with one of several drugs under trial. The drug for a given patient was selected by the participating physician with approval of the regional office. When entries for a given drug-tumour-type category became filled (the desired minimum sample size per category was 14 at the time), an alternate drug selection was requested. This is not the most desirable from a statistical standpoint but at the time it was the most workable administratively. Patients were examined routinely for toxicity and tumour measurements for at least 60 days. Treatment regimens varied according to the drug, from as short as 10 days for AB-132 to 60 days for 6-mercaptopurine (6-MP). Other drugs used by ECDEP were 5-fluorouracil (5-FU), chlorambucil and mitomycin-C.

Details of the protocols and criteria of response can be found in a report on 5-FU from the ECDEP (Moore *et al.*, 1968). Details of other drug regimens and toxicities etc. can be found in adjoining articles in the same issue of *Cancer Chemotherapy Reports*. Over 1600 acceptable patients were evaluated in this programme and 1432 had reliable information on the site of the measured lesions, totalling 2702. This is not an accounting of the total number of lesions but only those measured in the programme. Even patients with one measured lesion may have had multiple lesions which were not measured because of location or some other reason.

Though the majority of tumours being measured in this programme were metastatic, there were a fair proportion (19%) that were the original primary tumours. These were particularly prevalent in lung cancer (see Table I), with the primary representing half of the tumours. Because of the nature of this disease, only about 25% of new diagnoses are operable. In unoperable cases the primaries usually are preferentially treated with radiotherapy, then receive chemotherapy when they fail to respond to irradiation, hence their appearance in these data.

To evaluate the influence of metastatic site on tumour growth and response to chemotherapy independent from that of the primary site, it is necessary to have a group or groups of lesions representing several primary sites, each of which has tumours in the various metastatic locations. In other words, for the most informative and reliable analysis there should be a minimum of empty data cells in a cross-classification table of the variables under study. In these data more than one-third of the lesions were distributed among 23 of the less common primary sites, most of which did not have a representation of metastatic lesions in all the locations under study.

These have not been included in these analyses. Nearly two-thirds of the lesions were distributed among 10 of the primary sites. Even among these the distributions of metastatic sites required considerable collapsing to reduce the number of empty data cells.

Some of the primary sites were consolidated. These included a GI category composed of stomach, colon and rectum; and a GYN category composed of ovary, cervix and uterus. Other primary sites included in most analyses were mouth–pharynx–larynx (MPL) which were always recorded as one in the ECDEP, lung, breast, and melanoma. Consolidation of metastatic sites was also necessary. Categories used in most analyses consisted of lesions in the integumentary system, skeletal system, respiratory which was primarily lungs, haematological and lymphatic system which was primarily lymph nodes, and the digestive system which consisted mostly of liver and some epigastrium lesions. With this consolidation, the number of empty data cells was reduced to a minimum. It was impossible to eliminate them altogether and maintain a meaningful categorization for analysis. (Note the two empty cells in the overall distribution of metastases by primary site in Table I.) Several others occurred when the data were dicotomized into drug groups, all but two of which were eliminated by the further consolidation of " skeletal " and " digestive " metastases (see Table V).

The dependent variable used to represent lesion response to chemotherapy was the percentage change in cross-sectional area. All changes were determined by comparing the pretreatment measurements with the smallest size attained in the 60-day period. If the lesion never regressed, the largest size attained in 60 days was used.

Preliminary analyses were based on the " response rate " often used in the ECDEP reports, namely the percentage of patients whose tumours showed at least a 50% reduction in cross-sectional area. In subsequent analyses the " ridit " of the response was used since this takes into account the full range of size changes. The " ridit " analysis is explained further in the statistical appendix.

### RESULTS

### *Preliminary analysis*

A cross-classification of the more common primary sites and metastatic sites is shown in Table I. It is apparent from the distribution of metastatic sites within each primary site that there is a substantial difference in where metastases tend to appear. These distributions can-

TABLE I.—*Distribution of Major Areas of Metastasis for Some of the More Common Primary Sites*

| Metastatic system or site | Primary sites: MPL[a] No. | % | GI[b] No. | % | Lung No. | % | Breast No. | % | GYN[c] No. | % | Melanoma No. | % | Total |
|---|---|---|---|---|---|---|---|---|---|---|---|---|---|
| Integumentary | 115 | 54 | 71 | 16 | 25 | 19 | 173 | 40 | 56 | 20 | 107 | 57 | 547 |
| Skeletal | 7 | 3 | — | — | 4 | 3 | 63 | 15 | 7 | 3 | 5 | 3 | 86 |
| Respiratory[d] | 28 | 13 | 108 | 24 | 13 | 10 | 63 | 15 | 72 | 26 | 45 | 24 | 329 |
| Haemic and lymphatic | 35 | 17 | 26 | 6 | 20 | 15 | 47 | 11 | 23 | 8 | 7 | 4 | 158 |
| Digestive[e] | — | — | 143 | 32 | 2 | 1 | 18 | 4 | 15 | 5 | 5 | 3 | 183 |
| Primary[f] | 24 | 11 | 75 | 17 | 68 | 50 | 65 | 15 | 77 | 28 | 18 | 10 | 327 |
| Other | 2 | 1 | 22 | 5 | 3 | 2 | 4 | 1 | 24 | 9 | 2 | 1 | 57 |
| Total | 211 | | 445 | | 135 | | 433 | | 274 | | 189 | | 1687 |

[a] MPL = Mouth, pharynx, or larynx.
[b] GI = Stomach, large intestine, or rectum.
[c] GYN = Cervix, uterus, or ovary.
[d] Respiratory consists of lung primarily.
[e] Digestive consists of liver and epigastrium.
[f] " Primary " indicates the measured lesion was the primary lesion (as indicated across the top of table).

TABLE II.—*Number of Patients and Response Rate (50% Criterion) by Site or Origin and Site of Measured Lesion for Some of the More Common Sites*

| Site of measured lesion | Site of origin: MPL Pts | MPL %R | GI Pts | GI %R | Lung Pts | Lung %R | Breast Pts | Breast %R | GYN Pts | GYN %R | Melanoma Pts | Melanoma %R | Total Pts | Total %R |
|---|---|---|---|---|---|---|---|---|---|---|---|---|---|---|
| Integumentary | 115 | 25 | 71 | 32 | 25 | 52 | 173 | 24 | 56 | 25 | 107 | 23 | 547 | 27 |
| Skeletal | 7 | 14 | — | | 4 | 0 | 63 | 6 | 7 | 14 | 5 | 20 | 86 | 8 |
| Respiratory (lung) | 28 | 29 | 108 | 9 | 13 | 0 | 63 | 10 | 72 | 14 | 45 | 13 | 329 | 12 |
| Lymph nodes | 35 | 26 | 26 | 65 | 20 | 45 | 47 | 34 | 23 | 35 | 7 | 43 | 158 | 39 |
| Digestive (liver, epigastrium) | — | | 143 | 26 | 2 | 0 | 18 | 22 | 15 | 33 | 5 | 40 | 183 | 26 |
| Primary | 24 | 12 | 75 | 16 | 68 | 12 | 65 | 40 | 77 | 27 | 18 | 22 | 327 | 23 |
| Total | 209 | 24 | 423 | 23 | 132 | 23 | 429 | 23 | 250 | 24 | 187 | 22 | 1630 | 23 |

not be interpreted as representative of the true metastatic pattern because in the original trials of the ECDEP there was no effort made to record all lesions for a given patient. Skeletal lesions in particular were often not recorded because of the difficulty in obtaining reliable measurements. Nevertheless, it is apparent that if differences in growth and response at different metastatic sites exist, such divergent distributions among data series around the country could influence chemotherapy recommendations for some types of tumours. Superficial tumours in the integumentary system, for example, may be more responsive than other tumours. They comprised over half of all measured lesions for primary MPL tumours and melanomata, and 40% for primary breast tumours.

A general impression of the respective roles of the site of origin and the site of measured lesion can be obtained from Table II, which shows the distribution of patients over sites and the response rate in each cross-category. The response rate in Table II is the percentage of patients showing a 50% reduction in tumour size.

The response rates for the marginal totals of the rows and columns of Table II are based on fairly sizable series and tell a more coherent story than the response rates in the body of the table, since these are sometimes based on only a few patients. Glancing down the marginal totals for the rows, for the sites of the measured lesions it can be seen that there is a relatively wide range of response rates. For the overall responses to the 4 drugs under test from all sites of origins and all metastatic sites included in Table II, the response rate is 23%. The range for row totals is from a low of 8% and 12% for skeletal and respiratory lesions respectively, to a high of 39% for the lymph node lesions. This suggests that there are differential response rates for the different sites of the measured lesions.

By contrast, the response rates of the marginal totals for the columns, the sites of origin, are surprisingly uniform. They range only from a low for melanoma of 22% to a high for MPL and GYN of 24%. There is no suggestion in these totals that the site of origin has any important effect on the response rate. When the range of column rates is compared with the range of the row rates there is a clear impression that the site of the measured lesion has more influence on the response rates than the site of origin.

The results here appear to contradict a widely accepted chemotherapeutic doctrine that has long been the basis for the design of studies for the evaluation of new chemotherapeutic agents, particularly the Phase II studies based on metastatic lesions. In the ECDEP the analysis was based on site of origin and the inferences were drawn with respect to efficacy of drugs for particular sites of origin. Before calling into question an approach that has been used for many years, it is important to consider the possibility that there may be artefacts or other factors which are masking the effects of the site of origin in the marginals of Table II.

One possibility is that there are particular combinations of sites of origin and sites of measured lesion where the drugs tend to be effective or ineffective and that these happen to have balanced out for the columns and not in the rows. In other words, the result might be explained by a strong interaction between the two factors. There is some suggestion of an interaction in Table II, as can be seen by looking across the response rates in the rows. For example, the integumentary sites show a range from 23% to 52% with the highest response in tumours originating in the lung. The high value also stands out in looking down the column for lung as site of origin since the response rates (with one other exception) tend to be low. Another striking example of an interaction occurs in the row where the site of measured lesion is the primary and the range is from 12% to a high of 40% for tumours where the breast is the site of origin.

Another possible objection to accepting at face value the uniformity of the site-of-origin response rates in the column totals is that the results for all of the drugs tested are combined in Table II. This might be masking the importance of the site of origin if the response at different sites is markedly different for each drug. In other words, a strong interaction between drugs and site-of-origin might be masked by combining over drugs. Yet another objection might be that the argument so far has been impressionistic and based on casual inspection rather than a formal statistical analysis.

One way to meet these objections and to get further insight into the respective roles of the site of origin, site of measured lesion and drug in this complex interactive situation, is to use a somewhat more complicated type of statistical analysis. To take advantage of the entire range of response (rather than relying on an arbitrary 50% reduction criterion), the "ridit" of the response can be used to provide a more quantitative index. An analysis of variance gives a more formal approach to the testing of hypotheses about the interactions of the factors. The details can be found in the statistical appendix and the next section will present the main results.

One question of interest in its own right will be considered first: How do the response rates in the primary and the metastases from this site of origin compare? Overall results are shown in Table III. For some sites there are marked differences in response rates between the primary and the metastatic lesions, a point that raises some question about common practice of extrapolating from the latter to the former when Phase II studies are used to pick drugs

TABLE III.—*Proportion of Responses (50% Criterion) for Primary and Metastatic Tumours*

| Origin of primary | Primary tumours | | Metastatic tumours | |
|---|---|---|---|---|
| | R/T[a] | % | R/T | % |
| MPL | 3/24 | 12·5 | 47/187 | 25·1 |
| Stomach | 9/39 | 23·1 | 25/67 | 37·3 |
| L. Intestine | 3/25 | 12·0 | 47/186 | 25·3 |
| Rectum | 0/11 | 0 | 19/117 | 16·2 |
| Lung | 8/68 | 11·8** | 22/67 | 32·8 |
| Breast | 26/65 | 40·0** | 73/368 | 19·8 |
| Cervix | 11/45 | 24·4 | 17/85 | 20·0 |
| Ovary | 9/28 | 32·1 | 18/80 | 22·5 |
| Kidney | 0/4 | 0 | 12/96 | 12·5 |
| Bladder | 7/16 | 43·8 | 6/15 | 40·0 |
| Melanoma | 4/18 | 22·2 | 37/171 | 21·6 |
| Connective tissue | 4/27 | 14·8 | 31/184 | 16·8 |
| Undetermined | 1/7 | 14·2 | 43/184 | 23·3 |
| Total | 85/377 | 22·5 | 397/1807 | 22·0 |

[a] Responding (50% reduction) tumours over total number measured.

** Proportions significantly different from those of metastatic tumours for same site of origin ($P < 0{\cdot}01$).

for earlier stages of the disease. Because the response pattern of primary and metastatic lesions appeared different, the analysis of response ridits was done with this factor kept separate.

A number of other preliminary analyses were carried out on tumour response rates to determine what variables might be important for inclusion in the more detailed analysis of response ridits. Tumour size did not appear to be an important factor but the drugs used in the programme did appear to be important. Response patterns and levels were similar for mitomycin-C and 5-FU, and for AB-132 and chlorambucil; hence these two groups of two drugs each were used in the analysis. Data for 6-MP showed a different pattern of low level response with many missing cells, hence was not included.

*Analysis of response ridits*

The results of the analysis are summarized in the customary analysis of variance table (Table IV), and are discussed in terms that are as free of jargon as possible. The analysis of the measured lesions that were advanced primaries was performed separately (lines (L) 1–5 in Table IV) from that of the metastatic lesions (L 6–14 in Table IV).

The analysis of advanced primaries showed the various tumour types to have markedly different mean responses (significant F ratio in L 2), as might be expected. Mean values for the different tumour types were as follows:

| | Responsiveness of advanced primaries | | | | | |
|---|---|---|---|---|---|---|
| | MPL | GI | Lung | Breast | GYN | Melanoma |
| Mean ridit | 0·709 | 0·503 | 0·586 | 0·400 | 0·528 | 0·566 |
| Corresponding percentage reduction | 2% | 24% | 15% | 34% | 21% | 17% |

Fig.—Identified distribution for ridit calculations based on tumour response of 5-fluorouracil.

TABLE IV.—*Analysis of Variance of Response Ridits for Primary Measurement Sites and for Metastatic Measurement Sites*

| | Source of variation | Degrees of freedom | Sum of squares | Mean square | F ratio |
|---|---|---|---|---|---|
| | Analysis of primary measurement sites | | | | |
| L1 | Total | 279 | 23·4463 | | |
| L2 | Site of origin (O) | 5 | 1·5144 | 0·3029 | 4·10* |
| L3 | Drugs (D) | 1 | 0·0861 | 0·0861 | 1·17 |
| L4 | O × D | 5 | 2·0453 | 0·4091 | 5·54* |
| L5 | Within | 268 | 19·8004 | 0·0739 | |
| | Analysis of metastatic measurement sites | | | | |
| L6 | Total | 1113 | 96·3261 | | |
| L7 | Drugs (D) | 1 | 1·3705 | 1·3705 | 18·57* |
| L8 | Site of origin (O) | 5 | 0·7330 | 0·1466 | 1·99 |
| L9 | Site of measurement (M) | 3 | 3·2238 | 1·0746 | 14·56* |
| L10 | D × O | 5 | 1·9058 | 0·3812 | 5·16* |
| L11 | D × M | 3 | 0·0232 | 0·0077 | 0·10 |
| L12 | O × M | 15 | 3·8539 | 0·2569 | 3·48* |
| L13 | D × O × M | 15 | 6·5543 | 0·4370 | 5·92* |
| L14 | Within | 1066 | 78·6616 | 0·0738 | |

* Statistically significant effect ($P < 0·01$).

It may facilitate interpretation to convert these ridit values back to percentage reductions in tumour size (see Fig.). The above values convert to approximately a typical reduction of about 34% for breast primaries, 24% for GI primaries, and so on down to zero mean response for MPL primaries.

There was no significant overall effect of drugs, as grouped in this analysis, on primary tumour types but some tumour types responded differently on the 2 drug groups, as indicated by the significant interacting term in line 4. The mean response ridits for the different tumour types for each of the 2 drug groups are shown in the top section of Table V. Primary breast and gynaecological tumours responded best to AB-132 and chlorambucil while advanced primaries at the other sites responded best to mitomycin-C and 5-FU, though for neither drug group did the other sites (except GI) indicate promising activity. In fact, many of the metastatic sites responded better than these advanced primaries, as is evident from the mean responses in the bottom portion of Table V and as was evident from preliminary analyses discussed previously with Tables II and III. In summary, the analysis of the advanced primaries showed that the location of the primary had a marked effect on the mean response and indicated which drug showed promising activity.

The analysis of the metastatic tumours revealed that the metastatic (measurement) site influenced mean response strongly whereas the site of the primary from which the metastasis was derived did not. (Note the significant F ratio in line 9 and the non-significant ratio in line 8.) This effect can be seen by examining the " all sites " column and row in Table VI. Tumours metastatic to the more superficial areas such as the integumentary system and lymph nodes showed good mean responses, whereas those metastatic to deeper organ sites such as in the respiratory, skeletal or digestive systems showed poorer responses. The range of differences among these sites was considerably greater than among those for site-of-origin, although the variation seen in Table VI is not as good a measure of the actual difference in variation as the relative magnitude of the sums of squares in lines 8 and 9 of Table IV. The column mean for " lung " in particular is misleading unless

TABLE V.—*Mean Response Ridits of Site of Measurement by Site of Origin by Drug Group for Analysis of Primaries and of Metastases*

(Ridits × 1000)

| Drug group / Site of measurement | Site of origin: MPL | GI | Lung | Breast | GYN | Melanoma | All primary sites |
|---|---|---|---|---|---|---|---|
| Analysis of primary measurement sites | | | | | | | |
| Mito-C + 5-FU | 637 | 451 | 581 | 419 | 559 | 536 | 508 |
| AB-132 + Chlor. | 756 | 580 | 594 | 360 | 506 | 771 | 544 |
| Analysis of metastatic measurement sites | | | | | | | |
| Mito-C + 5-FU (Group A) | | | | | | | |
| Integumentary | 485 | 441 | 257 | 430 | 422 | 545 | 455 |
| Respiratory | 440 | 570 | 782 | 503 | 582 | 676 | 573 |
| Lymph nodes | 444 | 220 | 285 | 344 | 570 | 700 | 396 |
| Skeletal and digestive | 729 | 407 | 473 | 662 | 421 | 396 | 483 |
| All metastatic sites | 487 | 456 | 313 | 484 | 511 | 591 | 484 |
| AB-132 + Chlorambucil (Group B) | | | | | | | |
| Integumentary | 549 | 493 | 537 | 537 | 468 | 478 | 513 |
| Respiratory | 575 | 665 | 680 | 631 | 594 | 584 | 628 |
| Lymph nodes | 624 | 384 | 521 | 583 | 387 | — | 506 |
| Skeletal and digestive | — | 562 | 666 | 625 | 211 | 449 | 565 |
| All metastatic sites | 570 | 558 | 603 | 584 | 492 | 507 | 554 |

TABLE VI.—*Mean Response Ridits of Site of Measurement by Site of Origin*

(Ridits × 1000)

| Site of measurement | Site of origin: MPL | GI | Lung | Breast | GYN | Melanoma | All primary sites |
|---|---|---|---|---|---|---|---|
| Integumentary | 510 | 464 | 347 | 479 | 455 | 518 | 481 |
| Respiratory | 489 | 607 | 696 | 584 | 589 | 646 | 599 |
| Lymph nodes | 520 | 315 | 377 | 457 | 482 | 700 | 446 |
| Skeletal and digestive | 729 | 465 | 602 | 645 | 368 | 423 | 515 |
| All metastatic sites | 518 | 497 | 453 | 533 | 500 | 560 | 515 |

one takes into account the small numbers involved, as shown in both Tables I and II.

The mean response rates of metastatic tumours were markedly different for the 2 drug groups. The mean response ridits were 484 for mitomycin-C + 5-FU and 554 for AB-132 + chlorambucil, corresponding to typical reductions of about 25% and 15% respectively.

So much for the mean response of metastatic tumours: it depends far more strongly on where the metastasis is than on the site of the primary from which it originated. What, however, about the choice of which drug to use? Lines 10 and 11 of Table IV suggest that this depends almost entirely on the site of origin rather than on the site of the metastasis. This dependence can be seen by examining the 2 "all sites" rows in Table V. Four of the sites (MPL, GI, lung and breast) had better mean responses for mitomycin-C + 5-FU than for AB-132 + chlorambucil, while for the remaining sites, particularly melanoma, the opposite was true. Nevertheless, although this observation is consistent with the current aims of the Phase II drug screening programme, the highly significant D × O × M interaction in line 13 of Table IV warns us that the site of a metastasis does in fact have some relevance to the question of the drug to which it is likely to be most responsive. The interpretation of the large sum of squares in line 13 is moot. On the one hand, it could be due to the inadequacy of the additive linear model or to the data themselves, such as the non-randomness of the drug used. On the other hand again, it could be due to real effects. For example, in the bottom portions of Table V note that for tumours of MPL origin metastatic to lymph nodes or the respiratory system, the mean responses were good in the mitomycin-C + 5-FU drug group but poor in the AB-132 + chlorambucil group.

### DISCUSSION

It is apparent from these data that the mean response rate (as assessed by the percentage reduction in the size of a metastatic tumour after up to 60 days of chemotherapy) depends far more strongly on the site of the metastasis than on the site of the primary from which it was derived. This has important implications for both analysing data from and designing trials for the drug screening programmes. Depending upon the distribution of measured lesions in the sample of patients under trial, the proportion of responses, and hence the indication of drug activity for a given type of tumour, could be demonstrably affected. With a few exceptions, the superficial lesions such as lymph nodes and those in the integumentary system tended to be responders; and the deep lesions such as those in the skeletal and respiratory systems tended to be non-responders. Furthermore, it was apparent that the metastatic lesions that do respond do not give a reliable indication of drug action against parent tumour types.

This investigation was supported in part by the U.S. Public Health Service Research Grant No. CA-10378 from the National Cancer Institute.

### STATISTICAL APPENDIX

*Ridits.*—The dependent variable used in these analyses was obtained by converting lesion measurement changes to ridits. The term "ridit" stands for Relative to an Identified Distribution and may have a value from 0 to 1. The identified distribution (ID) can be any subseries or even the total, though most preferably a control series. In these analyses the distribution of lesion changes for 5-FU was used, since this drug has been tested on most tumour types and has been approved for general use. A detailed description of ridit calculations can be found in a paper by Bross (1958). Essentially they use the cumulative frequency distribution of the variable selected for the ID. In this case the maximum percentage change (relative to pretreatment measurement) in cross-sectional area for each lesion from patients treated with 5-FU, from complete regression (−100%) to the largest progression (+2050%), was used (see Fig.) and the ridits calculated. The conversion to ridits puts the entire range of response between zero and one and in a form that can be handled by conventional analyses.

### REFERENCES

Brennan, M. J., Talley, R. W., San Diego, E. L., Burrows, J. H., O'Brian, R. M., Vaitkevicius, V. K. & Horeglad, S. (1964) Critical Analysis of 594 Cancer Patients Treated with 5-Fluorouracil. In *Chemotherapy of Cancer*. Ed. Placidus A. Plattner. Amsterdam: Elsevier Pub. Co. p. 118.

Breur, K. (1966) Growth Rate and Radiosensitivity of Human Tumors. *Eur. J. Cancer*, **2**, 157.

Bross, I. D. J. (1958) How to Use Ridit Analysis. *Biometrics*, **14**, 18.

Moore, G. E., Bross, I. D. J., Ausman, R., Nadler, S., Jones, R. Jr, Slack, N. & Rimm, A. A. (1968) Effects of 5-Fluorouracil (NSC-19893) in 389 Patients with Cancer: Eastern Clinical Drug Evaluation Program. *Cancer Chemother. Rep.*, **52**, 641.

Rambert, P., Malaise, E., Langier, A., Schlienger, M. & Tubiana, M. (1968) Data on Growth Rate of Human Tumors. *Bull. Cancer, Paris*, **55**, 323.

Schneiderman, M. A. (1962) The Clinical Excursion Into 5-Fluorouracil. *Cancer Chemother. Rep.*, **16**, 107.